# Learning More May Not Be Better: Knowledge Transferability in Vision-and-Language Tasks

**DOI:** 10.3390/jimaging10120300

**Published:** 2024-11-22

**Authors:** Tianwei Chen, Noa Garcia, Mayu Otani, Chenhui Chu, Yuta Nakashima, Hajime Nagahara

**Affiliations:** 1 Institute for Datability Science, Osaka University, Osaka 565-0871, Japan; noagarcia@ids.osaka-u.ac.jp (N.G.); n-yuta@ids.osaka-u.ac.jp (Y.N.); nagahara@ids.osaka-u.ac.jp (H.N.); 2 CyberAgent Inc., Tokyo 150-0042, Japan; otani_mayu@cyberagent.co.jp; 3 Graduate School of Informatics, Kyoto University, Kyoto 606-8501, Japan; chu@i.kyoto-u.ac.jp

**Keywords:** vision and language, knowledge transferability analysis, multi-modal learning

## Abstract

Is learning more knowledge always better for vision-and-language models? In this paper, we study knowledge transferability in multi-modal tasks. The current tendency in machine learning is to assume that by joining multiple datasets from different tasks, their overall performance improves. However, we show that not all knowledge transfers well or has a positive impact on related tasks, even when they share a common goal. We conducted an exhaustive analysis based on hundreds of cross-experiments on twelve vision-and-language tasks categorized into four groups. While tasks in the same group are prone to improve each other, results show that this is not always the case. In addition, other factors, such as dataset size or the pre-training stage, may have a great impact on how well the knowledge is transferred.

## 1. Introduction

*The more data there are for learning, the better* seems to be the current *motto* in machine learning seems to be, as large language models achieve exceptional results on previously unseen tasks by being trained on hundreds of millions of samples crawled from the Internet [1,2,3,4]. Following the path led by natural language processing research, the computer vision community is gradually adopting Transformer-based models trained on web-scale datasets to achieve high performance in zero-shot settings [5,6]. This is conducted by leveraging huge amounts of image-caption pairs available online to let the models learn the correspondences between the language semantics and the visual appearance of objects.

The problem with using hundreds of millions of samples for training is that the analysis, maintenance, processing, and, particularly, understanding of the data are beyond human means. With rising concerns about large models encoding and perpetuating harmful representations towards historically discriminated groups [7,8], how data is handled acquires a crucial role. Knowing which datum is being used, why, and for what means is now more important than ever.

We try to answer *whether more data is always better* by systematically analyzing the transferability within vision-and-language tasks which is the subset of tasks that require both visual and language understanding to be solved, for example, image captioning [9] or visual question answering [10]. In the last decade, dozens of high-quality vision-and-language datasets were collected, cleaned, and used as de facto benchmarks for human-like reasoning [11,12]. Now, some of these datasets that were created with diverse motivations and purposes are coming together to train large vision-and-language models [13,14].

While some tasks can improve their performance when a model is trained on a multi-dataset and multi-task protocol [15], it is still unclear to what extent and whether all vision-and-language tasks can benefit from this. Our goal is to shed light on this question and explore the transferability of knowledge within vision-and-language tasks in a similar way as that in [16,17] for vision-only datasets. Specifically, we conducted hundreds of cross-experiments in which the performance of a target task trained under a dozen different initializations from different pre-trained source tasks were compared.

**Figure 1:**
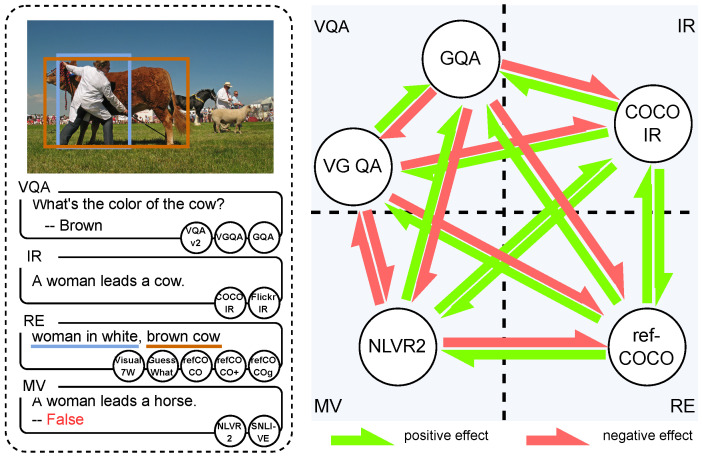
We explore the transferability among 12 vision-and-language tasks in 4 different groups: visual question answering (VQA), image retrieval (IR), referring expression (RE), and multi-modal verification (MV). Here, we illustrate the transferability among 5 tasks. Different tasks have different effects (positive or negative) on the other tasks.

Following [15], we divided vision-and-language tasks into four groups: visual question answering (VQA), image retrieval (IR), referring expression (RE), and multi-modal verification (MV). And we studied both intra- and inter-group transferability. As illustrated in Figure 1, our results indicate that there is not yet a magic formula to consistently improve performance on all the datasets by transferring knowledge between tasks. In other words, while some target tasks benefit from pre-training a specific source task, others are harmed. Even within target tasks that are similar in terms of datasets and goals, different behaviors are observed when the same source’s knowledge is transferred. Conversely, similar behaviors are seen when different knowledge is transferred. This leads to the conclusion that more data is not always necessarily better for higher performance since it depends on the training dataset’s goal, nature, and size.

From the experiments, we acquired several insights about the transferability of knowledge between vision-and-language models, which are summarized as follows:

- Tasks in the same group are more likely to help each other to improve performance. However, negative results show that tasks with shared goals do not always contribute positively to one another. This indicates that having a shared goal is favorable, but not enough.
- In the inter-group experiments, we find that the RE tasks tend to have a positive effect on most of the tasks in other groups, while the MV group tends to receive a positive effect from other groups.
- While the best improvement is often seen when knowledge is transferred within the same group, the worst results are concentrated on specific tasks, particularly GQA [18]. We study why and how this happens.
- We detect that different random seeds strongly affect the numeric performance of each task, sometimes even more than the transfer learning itself. This urges the reporting of vision-and-language results on multiple random configurations.
- We explore the effect of the data scale of the source task by downsampling a large-scale task. The results show increasing performance on all of the smaller-scale tasks, which indicates that the dataset size is an important but not always a positive factor in knowledge transferability.
- We also explore how different stages of training affect the performance of the target tasks. We discover that in some cases, transferring knowledge at the early stages of pre-training can benefit the target task. When the model learns too much, the performance on the target task drops.
- Finally, we analyze the similarity between the 12 tasks’ datasets and explore how the similarity between these datasets relates to knowledge transferability. We discover that tasks with different datasets can help each other, while tasks with similar datasets can bring negative effects. These results show that dataset similarity may not strongly affect vision-and-language tasks.

## 2. Related Work

Knowledge transferability focuses on how a model that learns knowledge from source tasks can adapt to a new task. Existing research on this topic includes transfer learning [5,19], multi-task learning [20,21], and meta-learning [22,23]. Ideally, the more knowledge a model learns, the better performance it has. However, in practice, models are affected by several phenomena, such as catastrophic forgetting [24,25], that limit their performance. Our work is mainly related to the following two topics:

### 2.1. Transferability Analysis

Transferability analysis studies how well the knowledge from a source task benefits a target task. Zamir et al. [16] proposed a method to analyze and utilize the transferability among 24 vision-only tasks on a single indoor-scene dataset. They pre-trained models in the source tasks, transferred them to the target tasks, and calculated the transferability by evaluating how well the model performed in the target task. Following this idea, Mensink et al. [17] studied the transferability between 20 real-world vision-only tasks. They analyzed three main factors: the image domain similarity between source and target tasks, the task type, and the data size.

While studies in [16,17] were conducted on vision-only tasks, we aim to explore multi-modality transferability in the vision-and-language domain. The particularity of multi-modal datasets is that knowledge needs to be transferred not only across tasks but also between modalities, which adds an extra layer of difficulty to the problem.

### 2.2. Paradigm of Solving Vision-and-Language Tasks

The most popular paradigm of knowledge transfer is to pre-train a model on a large dataset and transfer it to a downstream task [13,14,26,27,28,29,30,31,32,33]. For example, Lu et al. [13] proposed a BERT-based vision-and-language model, and pre-trained it with three self-supervised tasks to learn knowledge from Google’s Conceptual Captions dataset [34]. Following this work, many contributions were made in applying better text modeling [31], better visual feature extraction [32], and contrastive learning [6,14]. Furthermore, there has been some work analyzing the knowledge transferability in specific tasks such as video question answering [35].

Recently, CLIP [6] has shown a remarkable capacity to understand both vision and language data, by applying a specific image-text contrastive learning strategy. In this model, images and texts are separately processed by an image encoder and a text encoder, and the model is trained to match the image feature and text feature that belong to one pair. The specific design of CLIP makes it good for making zero-shot scenarios of vision-and-language tasks. Following CLIP, many studies explore how to utilize CLIP to improve models’ performances in existing vision-and-language tasks. Song et al. [36] explored the possibility of using the CLIP model directly for vision-and-language tasks in the scenario of few-shot learning. Tsimpoukelli et al. [37] and Shen et al. [38] explored the possibility of utilizing CLIP’s visual encoder and text encoder to extract more useful features for vision-and-language tasks. Li et al. [39] applied CLIP’s image–text contrastive learning strategy to the pre-training process of large vision-and-language models and explored how the training strategy benefits the pre-training model.

In general, our work is similar to CLIP in that we are also concerned about how downstream tasks can benefit from the pre-training. However, CLIP is different from us as we primarily focus on how the related tasks can help each other, while CLIP focuses more on how to train a model from an unrelated general dataset.

Our work is also close to multi-task vision-and-language learning [15,40,41,42]. Nguyen et al. [41] proposed a multi-task learning model with three vision-and-language tasks by choosing the best layers for each task. In [15], a training strategy to prevent learning too much knowledge from converged tasks is proposed, resulting in a model trained on 12 vision-and-language tasks. Following this idea, Hu et al. [42] designed a unified Transformer that can learn from either vision or text data. This model enables multi-task learning among vision-only, text-only, and vision-and-language tasks, and thus extends the knowledge that the vision-and-language model can learn.

None of the above authors conducted a formal analysis of how the different tasks affect each other. Conversely, we thoroughly explore knowledge transferability among vision-and-language tasks and uncover insights that may be useful when applying knowledge transfer methods to vision-and-language.

### 2.3. Vision-and-Language Tasks

This paper mainly explores knowledge transferability in four types of tasks: visual question answering [10,18,43], image retrieval [9,44], referring expressions [45,46,47,48], and multi-modal verification [49,50]. There are many other types of vision-and-language tasks, such as image captioning [9,51,52], text-to-image generation [4,53,54], and visual language navigation [55,56]. Furthermore, there are also other interesting tasks related to other modalities, such as video [57,58,59] and voice [60]. Evaluating more tasks could provide more insights about knowledge transferability, but we only focus on four types of tasks, following [15], that take both image and text as input.

## 3. Vision-and-Language Tasks in This Work

### 3.1. Visual Question Answering (VQA)

Given an image and a related question, VQA requires a model to select an answer from several candidates. The setting of VQA aims to not only explore the model’s capacity to understand both visual and linguistic data but also the capacity of knowledge reasoning, which is also known as a “visual Turing challenge” [61]. As the example in Figure 1 shows, when the VQA task gives the question “What’s the color of the cow?” that relates to the given image, the model not only needs to understand both the image and question but also check the color of the cow to give the proper answer “brown” to the given question. Beginning with the idea of the “visual Turing challenge”, Malinowski et al. proposed the classic “questing-to-image” formula and were the first to release the small-scale (about 12 K question-answer pairs) but workable dataset DAQUAR [61] for both training and evaluation. To solve the data-scale problem, Ren et al. generated question-answer pairs based on the COCO caption dateset [62] to construct the dataset COCOQA [62], which enlarges the scale of training data to about 82 K question-answer pairs. To further make a reliable dataset, large annotation projects [10,43,63] on visual question answering were launched and resulted in what are currently the most widely used datasets: VQA v2 [10] and Visual Genome QA (VG QA) [43]. They are summarized as follows:

When the standard visual question answering task shows the possibility for a model to answer visual questions, many studies start to explore the visual question answering models. For example, Hudson et al. proposed the GQA dataset that requires the model to focus more on the relations between visual contents. There are also studies that try to use visual question answering models to solve real-world challenges such as blind people caring [64].

In this paper, our exploration involves the following three VQA tasks: VQA v2 [10], VG QA [43], and GQA [18].

- **VQA v2** is a classic visual question answering task towards solving multi-modal problems. It contains 204 K images from MSCOCO [9] with 614 K human-annotated natural language question-answer (QA) pairs. In the evaluation process, models are required to predict one answer from all answer candidates around the whole dataset, i.e., each question has thousands of answer candidates.
- **Visual Genome QA (VG QA)** has a similar target as VQA v2, but has a larger dataset with 108 K images, 1.7 M QA pairs, as well as 5.4 M region descriptions and 2.3 M object relationships, which provide rich evidence for the analysis of visual question answering. Similar to VQA v2, this task requires the models to predict one answer from all the answer candidates around the whole dataset.
- **GQA** is more concerned with the models’ capacity on visual reasoning. It contains a dataset with 113 K images and 22 M QA pairs, which leverage the scene graph information from VG QA [43] to generate more challenging questions that need multiple reasoning steps to arrive at the answer. During the evaluation process, GQA also requires the models to predict one answer from all answer candidates around the whole dataset.

### 3.2. Image Retrieval (IR)

Given a caption, image retrieval requires the model to select the most representative image from a pool of images. The target of image retrieval is challenging as the images and sentences may be highly related to each other. Furthermore, image retrieval is also challenging when the task scale increases, as the time complexity of calculating the image-sentence matching score is about $O(n^{2})$. As shown in Figure 1, given the text “A woman leads a cow”, the model should find the related image as shown in the top-left part. The challenge of this task is that, although the image and sentence in one pair clearly match each other, many of the images and the sentences are very similar. The similarity between image-sentence pairs makes it difficult to distinguish the correct image by the given sentence. In the area of image retrieval, COCO IR [9] and Flickr30K IR [51] are two of the most widely used datasets, which are both for exploring models’ capacity to retrieve correct images. In recent years, many valuable challenges have been proposed in the formula of image retrieval, such as artwork retrieval [65] and food retrieval [66].

In this paper, our exploration involves the following two IR tasks: COCO IR [9] and Flickr30K IR [44]. Both are summarized as follows:

- **COCO IR** in an image retrieval task based on the COCO caption dataset [9]. In this task, there are 123 K images with 567 K related human-annotated captions. To evaluate the models’ performances, COCO IR provides three accuracy scores with different recall scales: the accuracy on top one retrieval (Recall@1), the accuracy on top five retrievals (Recall@5), and the accuracy on top ten retrievals (Recall@10). In this paper, we use Recall@5 as the main metric for the evaluation.
- **Flickr30K IR** is an image retrieval task based on the Flickr30K dataset [44]. It has 31 K images with 146 K human-annotated captions. Similar to COCO IR, Flickr30K IR uses Recall@1, Recall@5, and Recall@10 to evaluate the models’ performances. In this paper, we also use Recall@5 as the main evaluation metric.

### 3.3. Referring Expression (RE)

Referring expression concerns the relation between linguistic expressions (i.e., texts) and visual contents (i.e., objects), which can be divided into two directions: (1) detecting visual contents based on the expressions, or (2) generating expressions by the given visual contents. In this paper, we mainly focus on the first direction of referring expression. Given a text and an image, referring expressions require the model to detect the corresponding region in the image described by the text. In contrast to image retrieval, referring expressions do not focus on retrieving one image from a group. Instead, it focuses on detecting the related region from one image. Thus, compared to image retrieval, referring expression is more concerned about the specific objects in one image. As the example in Figure 1 shows, instead of the text about the whole image (e.g., “A woman leads a cow”), texts such as “woman in white” and “brown cow” are given as the targets, which are related to certain objects. Referring expression is used to be a classic natural language processing task that has been studied since the 1970s [67]. Since then, researchers have been interested in how models can work as humans to link texts and visual contents and add more challenges, such as applying real-world images [47,48], enriching object categories [46], and increasing the level of difficulty in visual reasoning [45].

In this paper, our exploration involves the following RE tasks: Visual7w [45], GuessWhat [46], and RefCOCO(+/g) [47,48]. They are summarized as follows:

- **Visual7w** is a referring expression task based on the Visual 7w dataset [45]. In this task, there are 25 K images with 151 K region-text pairs. The evaluation method is the accuracy achieved if a model predicts a region that has an Intersection over Union (IoU) score higher than 50%.
- **GuessWhat** is a referring expression task based on the GuessWhat dataset [46]. In this dataset, there are 66 K images with 137 K region-text pairs. The same as Visual7w, GuessWhat evaluates models’ performances by the object prediction accuracy with IoU >50%.
- **RefCOCO(+/g)** [47,48] are three similar referring expression tasks that leverage the image and object information from the COCO dataset [9]. Among these tasks, refCOCO and refCOCO+ are collected by ReferitGame [47], which is an interactive game between two players as one player expresses an object and the other player points it out. refCOCO+ is more challenging than refCOCO as it restricts the player by not allowing them to use location words during the game, while refCOCOg [48] collects its data in a non-interactive setting, which asks annotators to express the given object directly. In general, refCOCO has 19 K images with 131 K expressions, refCOCO+ has 19 K images with 130 K expressions, and refCOCOg has 25 K images with 90 K expressions. The same as the above referring expression tasks, refCOCO(+/g) evaluates the models’ performances using the object prediction accuracy with an IoU >50%.

### 3.4. Multi-Modal Verification (MV)

Given one or more images and a referred text, multi-modal verification requires the model to decide whether the text is correct or not. Different from the rest of the three groups of tasks, multi-modal verification tasks usually have a very limited number of answers, e.g., NLVR2 [49] only has two candidate answers, and SNLI-VE [50] only has three candidate answers. However, multi-modal verification is challenging in the sense that it requires more visual reasoning capacity to verify if the text and image conflict. As shown in the example in Figure 1, multi-modal verification requires the model to judge if “a woman leads a horse” in the image. The verification is considered challenging as understanding “a woman” and “a horse” cannot directly lead to the correct answer. The idea of recent multi-modal verification studies, such as CLEVR [68,69], is motivated by the progress of visual question answering, which shows the evidence that deep learning models are capable of making knowledge reasoning. The results of these two tasks are encouraging and further motivate the tasks of NLVR2 [49] and SNLI-VE [50], which are currently the most widely studied multi-modal verification tasks.

In this paper, our exploration involves the following two MV tasks: NLVR2 [49] and SNLI-VE [50]. They are summarized as follows:

- **NLVR2** [49] is a multi-modal verification task that requires models to verify if one sentence is true in two images, i.e., the task takes one text and two images as the input and requires the models to give a binary answer (true or false). This specific design is to verify if the model can present a reasoning across not only the modality but also different data in the same modality. The dataset of this task contains 103 K real-world images with 93 K human-annotated texts. The evaluation process involves calculating the accuracy of the models’ binary predictions.
- **SNLI-VE** [50] is another multi-modal verification task to verify if a hypothesis (text) is accurate (entailment), partly accurate (neutral), or wrong (contradiction) to a given premise (image). The dataset of this task contains 31 K images with 548 K texts, and the evaluation metric is the accuracy of the triplet classification.

## 4. Methodology

**Figure 2:**
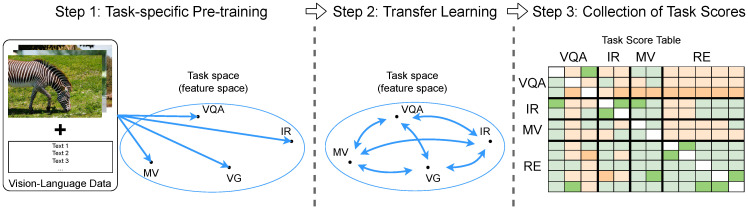
Analysis of transferability relationships between tasks. In Step 1, we trained 12 vision-and-language tasks independently. In Step 2, we used the models from Step 1 and fine-tuned them on each of the other tasks. In Step 3, we formed a transferability relation table for the 12 vision-and-language tasks divided into 4 groups: visual question answering (VQA), image retrieval (IR), multi-modal verification (MV), and referring expression (RE).

We studied how the knowledge from a source task affects a target task. Formally, we define the problem as follows:

Given a set T of vision-and-language tasks, we pick out a source task s∈T and a target task t∈T. We train a *direct* model $m_{t}$ by training a model *m* with the target task *t*. We also train a *one-hop* model $m_{s\to t}$ by pre-training *m* with *s*, and then with *t*. As shown in Figure 2, the performances of a pair of models $\left(m_{t},m_{s\to t}\right)$ are compared for all possible combinations of *s* and *t* in T. Tasks are categorized into groups according to their main goal, so tasks with similar goals are assigned to the same group.

### 4.1. Tasks Selection

As introduced in Section 3, we studied 12 vision-and-language tasks categorized into 4 groups. The feature of each task is listed in Table 1. Please note that the number of samples and images in the Train + Val set is the number after the cleaning in Section 5.

### 4.2. Model

We followed the model structure in [15], consisting of a unified multi-modal encoder based on VilBERT [13] with 12 different task-specific heads for corresponding tasks. The training goal is

(1) $$\arg\min_{\theta_{e},\theta_{t}}L_{t}\left(\psi_{\theta_{t}}\left(\phi_{\theta_{e}}\left(V_{t},S_{t}\right)\right)\right),$$

where $V_{t}$ and $S_{t}$ are the image and text in the dataset of task *t*, and $\theta_{e}$ and $\theta_{t}$ are the parameters of the encoder ϕ and the task *t*’s head ψ, respectively. $L_{t}$ is the loss of the task *t*.

### 4.3. Workflow

The workflow, as shown in Figure 2, was split into three steps: (1) task-specific pre-training, (2) transfer learning, and (3) collection of scores.

*Task-specific pre-training.* We pre-trained each of the 12 tasks independently, i.e., each task s∈T was trained by its corresponding dataset and did not see any datasets from other tasks. We collected the trained models $m_{s}$ from each task as the pre-trained models, which learned task-specific knowledge from the source task. We also evaluated each *direct* model $m_{t}$ as baselines for non-transferred knowledge.

*Transfer learning.* We fine-tuned, again, each pre-trained model $m_{s}$. Given $m_{s}$ and the target task *t*, we obtained a final model $m_{s\to t}$ by fine-tuning $m_{s}$ with all of the training samples in task *t*.
*Collection of scores.* We categorized tasks into groups and evaluated all *direct* models $m_{t}$ and *one-hop* models $m_{s\to t}$ for all possible task pairs. The results are discussed in Section 5.2.

## 5. Experiments on VilBERT

*Datasets.* We used the same set of datasets as [15], including the training and test sets of the 12 tasks. The overlapping samples from the different tasks were removed from the training sets to prevent data leaking from the test set into the training set. Note that the original test sets were not changed during this cleaning process. For the training and validation sets, VQA v2, VG QA, COCO IR, and NLVR2 had about 100,000 images; GQA and GuessWhat had about 60,000 images; Flickr30K IR and SNLI-VE had about 30,000 images; and refCOCO, refCOCO+, refCOCOg, and Visual7w had about 15,000 images.

*Experimental settings.* We followed most of the settings in [15]. We modified the batch size to 1/4 to fit the training in our server. (We used a single server with 4 16 GB NVIDIA P100 GPUs.) Pre-trained models $m_{s}$ were trained for 6 epochs, which was enough for convergence. To ensure that the models $m_{s\to t}$ learned task-specific knowledge well, we used the models with the best performance in the validation set, except for VG QA, which was evaluated on the validation set, and thus, the model at the 6th epoch was used. All of the models were seen to converge into their corresponding tasks. We trained every model with three different random seeds and reported the results by their mean and standard deviation.

*Evaluation metrics.* We used accuracy for tasks in the VQA group and the MV group. For the IR group, we used Recall@5. For the RE group, we followed [15,47,48] and computed the score based on the Intersection over Union (IOU) between the ground truth and the prediction.

### 5.1. Random Seed

**Figure 3:**
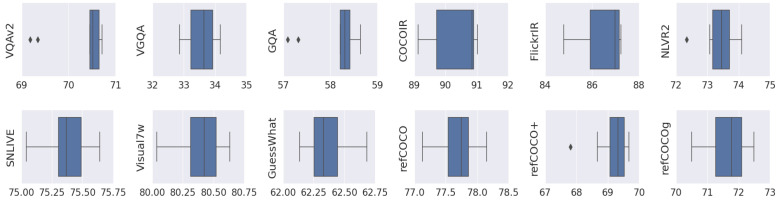
Box plots of the 12 tasks trained with 10 random seeds showing a big gap between the best and the worst scores.

Preliminary results showed large variations in performance when models were trained under different random initializations, as was also shown in [71]. Thus, before proceeding with the transferability experiments, we first explored the instability of vision-and-language tasks and their sensibility to random seeds. We trained each direct model, $m_{t}$ for all t∈T, 10 times with different random seeds. The results are shown in Figure 3. Although most of the tasks presented a gap larger than 1% between the maximum and the minimum scores, most of the scores in each task were concentrated in a small region. More details are shown in the box plots in Table 2. Nine tasks had a gap larger than 1%. Among them, Flickr30K IR was the one that fluctuated the most, with a gap of 2.44% and a standard deviation of 0.87. This revealed that experiments on a single run may not be reliable enough to extract conclusions about model performance. In general, we found that the random seed had a big impact on the evaluation of vision-and-language tasks. To ensure that our results were reliable, we ran each experiment three times.

### 5.2. Results by Group

For the transferability experiments, we collected results from 12 *direct* models $m_{t}$ and 132 *one-hop* models $m_{s\to t}$ and present them herein in Table 3. We used the results of $m_{t}$ (Row “*direct* model $m_{t}$”) as the baseline. We relied on a color scheme to illustrate the comparative performance of the transferred models $m_{s\to t}$: deep green for the best scores of each column, i.e., the best results of each task in transfer learning, and deep orange for the worst results. For the rest of the entries of the tables, light green and light orange indicated better and worse performance than the baseline, i.e., positive or negative transfer of the knowledge.

#### 5.2.1. Visual Question Answering Group

Columns 1–3 (VQA v2, VG QA (Val), and GQA) in Table 3 show the results in the VQA group. VQA v2 and GQA benefit from each other, but they do not improve the VG QA performance. In fact, GQA has the worst effect on VG QA among the 12 tasks. VG QA achieves its best performance with the help of refCOCOg, indicating that even though it is commonly seen as a VQA task, it may be closer to the RE group. When tasks in the VQA group are the target tasks, the source tasks have a consistent effect on each of them, e.g., COCO IR gives the best effect to VQA v2, while giving a negative effect to both VG QA and GQA. In contrast, GuessWhat gives the worst effect on GQA, while giving a positive effect on VQA v2. More specifically, VQA v2 and VG QA show contrary behavior: VQA v2 obtains a positive effect from all of the tasks outside the VQA group, while only refCOCO and refCOCO+ give VG QA a positive effect. This indicates that although VQA, VG QA, and GQA have the same type of training goal, their underlying knowledge may be very different, and thus receive different contributions from the same source task. Finally, even though tasks in the VQA group are the largest in terms of training samples when they act as the source task, they tend to have a negative impact on the other group tasks (Row 1–3 in Table 3), indicating that large training sets are not a guarantee for a better transfer.

#### 5.2.2. Image Retrieval Group

Columns 4–5 (COCO IR and Flickr30K IR) in Table 3 summarize the performance of the IR group. Both tasks in this group help each other. Also, as source tasks, they show similar behavior, with a tendency to improve other tasks. However, the results in this group show the largest variance. On one hand, as target tasks, only the VQA group has a consistently negative impact on Flickr30K IR. On the other hand, the standard deviation scores in COCO IR and Flicker30K IR are usually larger than in other groups. The standard deviation scores of $m_{\mathrm{COCO}\;\mathrm{IR}\to \mathrm{Flickr}30K\;\mathrm{IR}}$ and $m_{\mathrm{Flickr}30K\;\mathrm{IR}\to \mathrm{COCO}\;\mathrm{IR}}$ tend to be larger than tasks in other groups.

#### 5.2.3. Multi-Modal Verification Group

Columns 6–7 (NLVR2 and SNLI-VE) in Table 3 list the performance of the MV group. Except for GQA, most of the source tasks have a positive effect on the two MV tasks. NLVR2 and SNLI-VE also improve each other, but the effect is not larger than the ones from COCO IR and refCOCO+. This may be in part because NLVR2 and SNLI-VE are considerably different: NLVR2 is a binary classification task that verifies if a comment describes a fact among multiple images, while SNLI-VE is a ternary classification task that verifies how well a comment describes an image. Another reason may be because of the data distributions: NLVR2’s images are from ILSVRC 2014 [72], while SNLI-VE’s are from Flickr30K [51].

#### 5.2.4. Referring Expression Group

Columns 8–12 (Visual7w, GuessWhat, refCOCO, refCOCO+, and refCOCOg) in Table 3 lists the scores of the RE group. All the tasks in this group benefit from transferred knowledge in the same group. The improvements within this group, especially among refCOCO, refCOCO+, and refCOCOg, are larger than those from tasks in other groups. However, all tasks in the VQA and the MV groups have a negative effect on the RE group (except $m_{\mathrm{NLVR}2\to \mathrm{refCOCOg}}$). RE tasks also receive the worst effect from the GQA task. Tasks in this group usually have a positive outcome on the tasks in other groups, according to rows 8–12 in Table 3. This may be because of the nature of the group: RE tasks aim to find image regions given a text, which can be helpful to VQA, IR, and MV.

### 5.3. Main Observations

**Observation** **1.** **Intra-group analysis: tasks in the same group tend to improve each other, but not always.** Tasks in the IR, MV, and RE groups help other tasks in the same group to achieve better performance. However, tasks in the VQA group show a different behavior: only half of the intra-class relationships are positive. This indicates that (1) the defined task groups based on shared goals may be superficial and not a good representation of the internal type of knowledge in each task, and (2) having a shared goal may be favorable, but it is not enough for successfully transferring knowledge between tasks.
**Observation** **2.** **Inter-group analysis: some groups are more prone to help, while others are a disservice.** For example, while tasks in the RE group usually give a positive effect on most of the tasks that are in other groups, tasks in the VQA group produce no benefit to the tasks in the RE group, and only one task in the MV group (NLVR2) gives a slightly positive effect to a task in the RE group (refCOCOg). This indicates that the knowledge in certain groups, such as RE, may be more general, and thus easier to transfer, than task-specific knowledge from other groups, e.g., VQA.
**Observation** **3.** **Benefits in knowledge transferability are not reciprocal.** For example, VQA v2 receives a positive effect from all of the other 11 tasks, but it contributes negatively to most of these tasks, except GQA, NLVR2, and SNLI-VE. The same happens between the MV and RE groups. RE consistently improves the MV group, including the best effect on SNLI-VE from refCOCO+. However, the MV group harms all the tasks in the RE group except $m_{\mathrm{NLVR}2\to \mathrm{refCOCOg}}$. This is consistent with the observations in [15].
**Observation** **4.** **The best effect tends to come within the group, while the worst effect is usually from GQA.** The best results for each task are usually from a source task in the same group, which reinforces the idea that tasks with the same target tend to benefit each other more (Observation 1). The worst results, however, are usually caused by GQA. Many reasons may cause this, for example, the difference in the data scale between GQA and the rest of the tasks or the knowledge for solving GQA may be too specific. To better understand the phenomena, we conducted additional experiments and present them in Section 5.4 and Section 5.5.

### 5.4. Data Scale

Next, we investigated the effect of the data scale on the transferability of knowledge. As discussed in Section 5.3, GQA pre-training tends to harm many of the rest tasks. We speculated that one of the reasons may be because GQA has a much larger training set than the other tasks. To investigate this hypothesis, we used GQA as the source task and downsampled its training set from 962,928 to 96,221, which is close to the scale of seven tasks: refCOCO, refCOCO+, refCOCOg, Visual7W, GuessWhat, NLVR2, and Flickr30K IR. We pre-trained models with the reduced and full GQA training sets. The full GQA model and the reduced GQA model were then trained again on the above seven tasks in the same way as in Section 4.3. We also compared them against their direct models.

**Figure 4:**
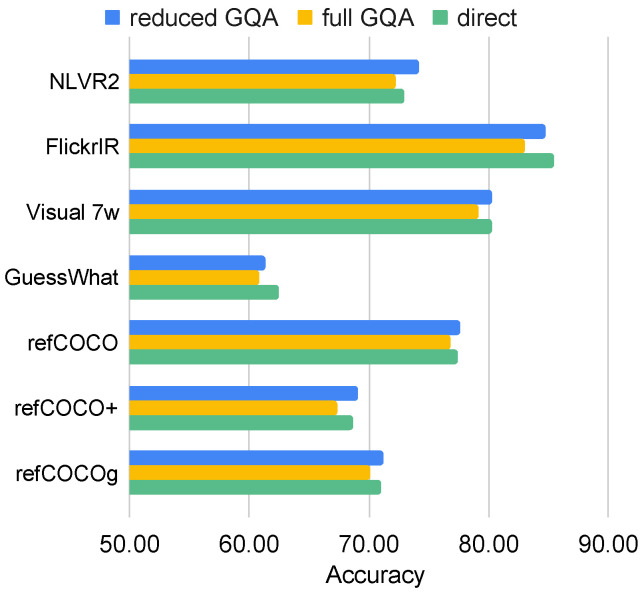
Accuracy of seven tasks pre-trained with a smaller set of GQA (reduced GQA), the full set of GQA (full GQA), and without pre-training (direct).

Figure 4 shows the accuracy of these seven tasks pre-trained on the reduced GQA, the full GQA, and the direct models. All models pre-trained on the reduced GQA achieved better performance than those pre-trained on the full GQA, which indicates that the data scale is a crucial factor in the transferability. When comparing the models derived from the reduced GQA with the direct models, the reduced models improved the performance of four of the seven tasks (NLVR2, refCOCO, refCOCO+, and refCOCOg), showing that GQA can also contribute positively as a source task. The results show some similar phenomena to [73], as the large data scale may not necessarily generate better results.

### 5.5. Training Epoch

We finally explored the relationship between the number of training epochs of the source task and the success of the knowledge transferred in the target task. We conjectured that, for a target task that receives a negative effect from a source task, the more a model learns from the source task, the worse the model performs on the target task. To verify this, we used GQA as the source task, which tended to give the most negative effect to the other tasks. Furthermore, we chose refCOCO and NLVR2 as the target tasks, which obtained the worst performance from GQA. We obtained six pre-trained $m_{\mathrm{GQA}}^{e}$ models, where the number of epochs was e={1,⋯,6}. The higher the epoch, the more knowledge $m_{\mathrm{GQA}}^{e}$ learns from GQA. We transferred these models to refCOCO and NLVR2.

**Figure 5:**
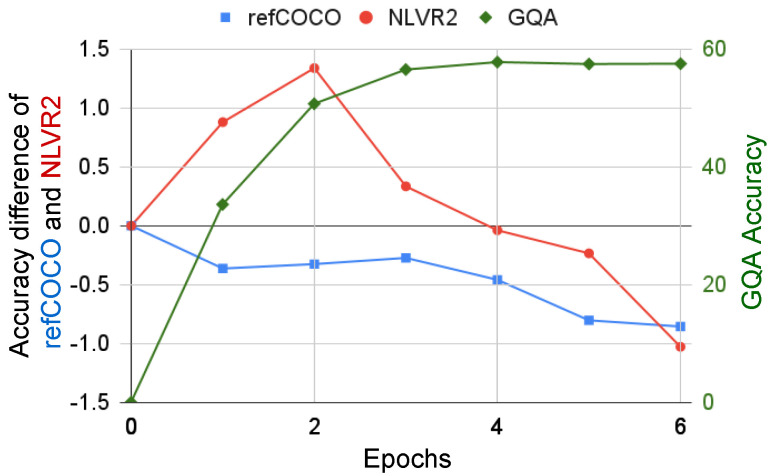
Accuracy of refCOCO (▪) and NLVR2 (•) fine-tuned with $m_{\mathrm{GQA}}$ after different epochs of pre-training. As a reference, the accuracy of GQA (⧫) is also shown.

The results are illustrated in Figure 5. The blue ▪ and red • are the scores for refCOCO and NLVR2, respectively, which, for visibility, are shown as the difference with respect to the direct model, i.e., $a={\mathrm{Acc}}_{\mathrm{GQA}\to t}^{\left(e\right)}-{\mathrm{Acc}}_{\mathrm{GQA}}$, where ${\mathrm{Acc}}_{\mathrm{GQA}\to t}^{\left(e\right)}$ is the accuracy of the model pre-trained with GQA for *e* epochs and fine-tuned with task *t*; ${\mathrm{Acc}}_{\mathrm{GQA}\to t}^{\left(0\right)}$ is the model that has no training on GQA, i.e., the direct model; and *t* is either refCOCO or NLVR2. For comparison, we also show the GQA accuracy (${\mathrm{Acc}}_{\mathrm{GQA}}^{\left(e\right)}$, green ⧫). Both tasks obtained lower scores than the direct model when using a model trained on GQA for more than four epochs. In the case of refCOCO, it achieved an inferior performance in all training epochs. In contrast, NLVR2 improved by more than 1% from GQA pre-trained for two epochs, showing that the knowledge from GQA does not always have a negative effect.

### 5.6. Data Domain Similarity

#### 5.6.1. Data Domain Distance

We explored how the similarity of the data domain affects the transferability between vision-and-language tasks. On the one hand, some tasks take images from the same image dataset, e.g., images in VQA 2.0, COCO IR, Visual7W, and GuessWhat are all from the MSCOCO [74] dataset. On the other hand, some tasks (e.g., refCOCO, refCOCO+, and refCOCOg) have similar text data. Intuitively, two tasks with similar data domains may face smaller domain shifts when fine-tuning and thus tend to have a higher probability of helping each other. To explore this, we randomly took 1000 samples from the training set of each task, used VilBERT [13] to extract the features from the samples, and calculated the domain distance similarly to Mensink et al. [17]:

(2) $$D\left(t|s\right)=\frac{1}{\left|t\right|}\sum_{z_{t}}\left(\underset{z_{s}}{min}\;d\left(f_{z_{t}},f_{z_{s}}\right)\right),$$

where d() is the Euclidean distance, and $z_{s}$ and $z_{t}$ are the samples from source tasks *s* and *t*, respectively. Please note that VilBERT [13] is not pre-trained on any of the 12 tasks.

**Figure 6:**
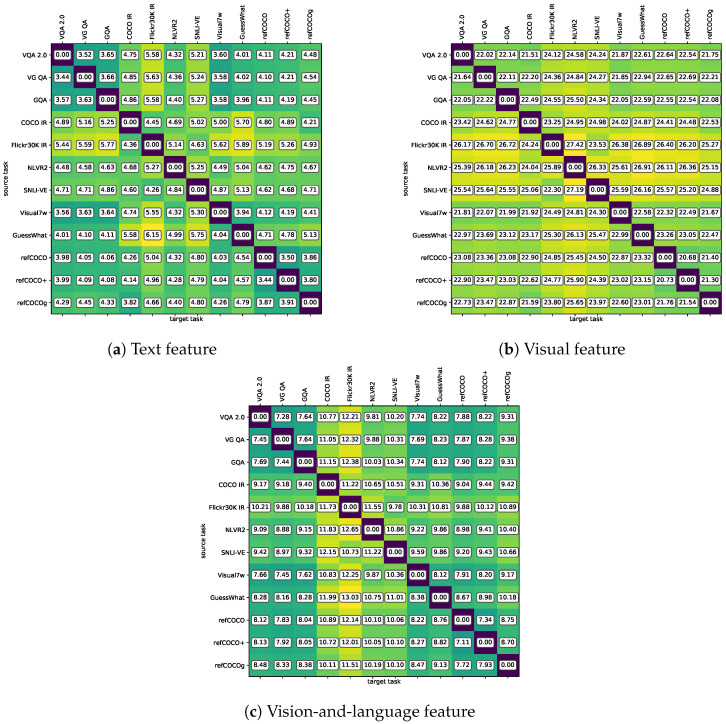
Domain distance between 12 vision-and-language tasks. We calculated the distances of the vision-and-language feature (i.e., fused feature), text feature, and visual feature. Each of the blocks shows the domain distance of $D_{row\;\to \;column}$. Please note that the distance of $D_{row\;\to \;column}$ and $D_{row\;\to \;column}$ may not be the same, as (2) finds the closest source task sample for each target task sample.

In contrast to the analysis of vision-only tasks in [17], vision-and-language tasks concern the visual and text data as well as the hidden relation between both data. Thus, our domain distance exploration involves the analysis of the vision feature, text feature, and fused vision-and-language feature, respectively. The results are illustrated in Figure 6.

In general, on the one hand, some tasks are close to other tasks from the data domain view, e.g., refCOCO, refCOCO+, and refCOCOg are close to each other in all three figures. The close distance of these three tasks is because of the same image origination and the similar text collection process. Since refCOCO, refCOCO+, and refCOCOg are in the same data scale, the close distance of the data domain becomes one of the factors that these three tasks can help each other to achieve a better performance.

Figure 6c illustrates that Flickr30K IR has a larger distance as the target task compared with other tasks. This indicates that the data from other tasks are more different from Flickr30K IR, and the transfer learning toward Flickr30K IR may bring fewer benefits. As shown in Column 4–5 in Table 3, while having the same data scale as Flickr30K IR, GuessWhat and NLVR2—the largest and the second largest distances from Flickr30K IR—show a negative effect on Flickr30K IR.

However, the domain distance is not as strong a decisive factor as the data scale. Figure 6c illustrates that GQA is not much different from most of the other tasks. Instead, GQA has the top-5 closest data domains to NLVR2, Visual7W, GuessWhat, refCOCO, refCOCO+, and refCOCOg. However, GQA causes the worst results for these tasks. This fact indicates that the data scale may have a more decisive effect on knowledge transferability.

#### 5.6.2. Appearance Distribution

**Figure 7:**
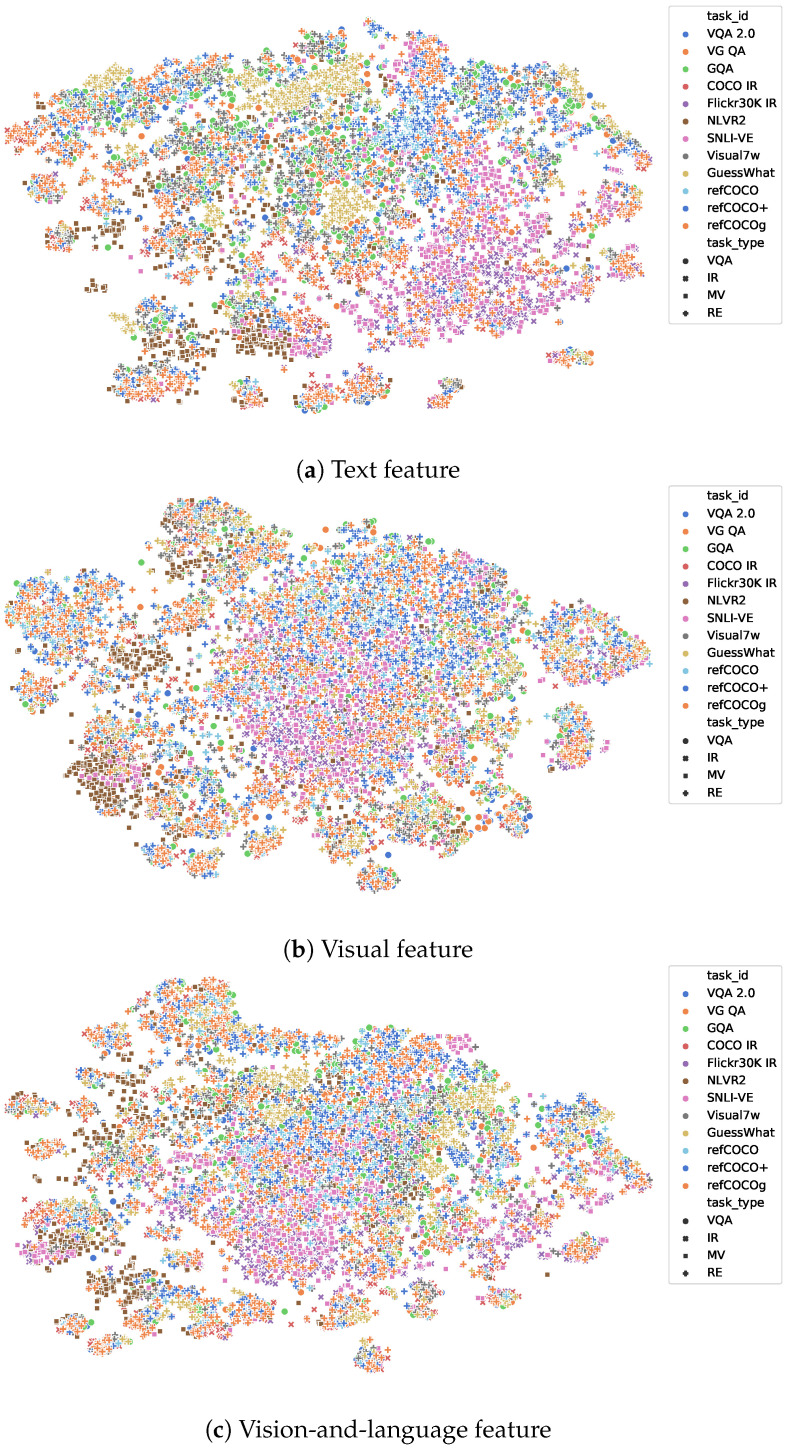
Appearance distribution of all 12 vision-and-language tasks. In this figure, different tasks are given different colors while tasks within the same task group share the same shape of markers.

**Figure 8:**
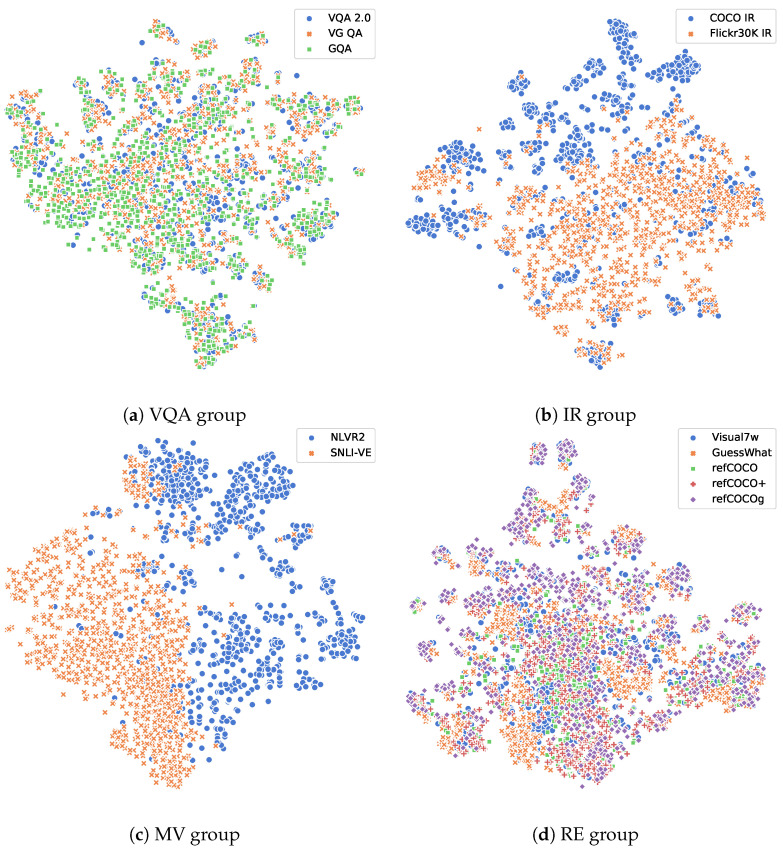
Appearance distribution within four vision-and-language task groups. In this figure, different tasks are given different colors and different shapes of markers.

Furthermore, we explored the data domain distribution in the embedding feature space, namely, the appearance distribution of all 12 tasks. The analysis was based on the sample feature in the last experiments, i.e., we randomly took 1000 samples from the training set of each task and used VilBERT [13] to extract the features from the samples. To make a better visualization of the distribution, we used t-SNE to plot the samples into 2D figures, as shown in Figure 7 and Figure 8.

Figure 7 illustrates the data appearance distribution of all 12 tasks. We find that in many cases, a sample from one task may be closer to the samples in another task, even though the two tasks have different data origination (refer to Table 1) (e.g., although NLVR2 (the brown squares) and VQA 2.0 (the blue dots) have different data origination, their samples are closed to each other). This indicates that the 12 tasks have a close appearance distribution, and they are very similar to each other.

The similarity of data distribution not only appears in the vision-and-language feature (Figure 7c) but also in the vision-only feature (Figure 7a) and text-only feature (Figure 7b). This indicates that the 12 tasks are similar to each other from both the image and text domains.

Figure 8 further shows the appearance distribution of vision-and-language features for each task group. On the one hand, tasks in the VQA group and RE group have similar appearance distribution to other tasks in the same group, which indicates that tasks in these task groups are similar from the data domain view. On the other hand, tasks in the IR group and MV group have different appearance distribution from each other, which indicates that the data of these tasks are different from each other and the knowledge from one task may not be much help to the other task. However, we can still observe from Table 3 that COCO IR and Flickr30K IR help each other to obtain the best performance, and NLVR2 and SNLI-VE help each other to improve. These results show that task similarity may be more decisive than data domain similarity in improving the model performance, i.e., task similarity affects knowledge transferability more than data domain similarity.

### 5.7. Visual Results

**Figure 9:**
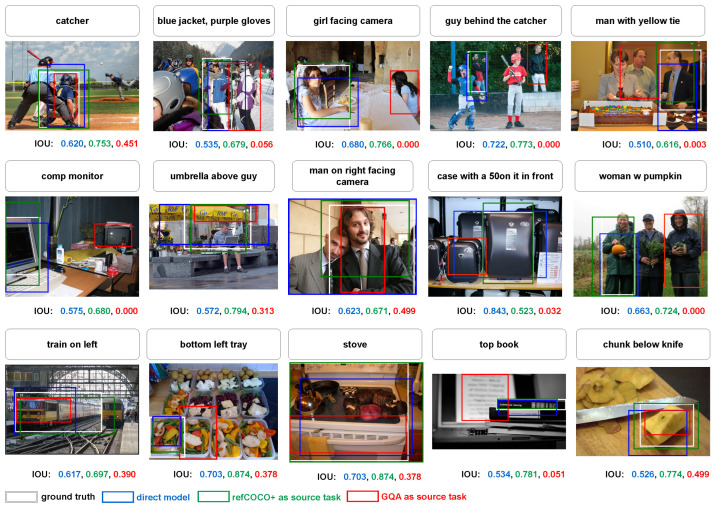
Example of the results on refCOCO. With the caption on top of the image, different models find different regions on the image. It is easy to see that refCOCO+ helps refCOCO to obtain a more accurate prediction, while GQA misleads refCOCO to some wrong regions.

Figure 9 shows predictions on refCOCO with the direct model $m_{\mathrm{refCOCO}}$, one-hop model $m_{\mathrm{refCOCO}+\to \mathrm{refCOCO}}$, one-hop model $m_{\mathrm{GQA}\to \mathrm{refCOCO}}$, and the ground truth. The IOU between the prediction and the ground truth is shown under the respective image. Whereas refCOCO+ helps to find more accurate regions and to obtain higher IOU, GQA misleads the task to smaller or even wrong regions. For example, in the image in the middle, although the direct model finds the region with the right person, refCOCO+ helps to find a more accurate region, but GQA predicts the wrong person. The same behavior can be observed in the last two images.

**Figure 10:**
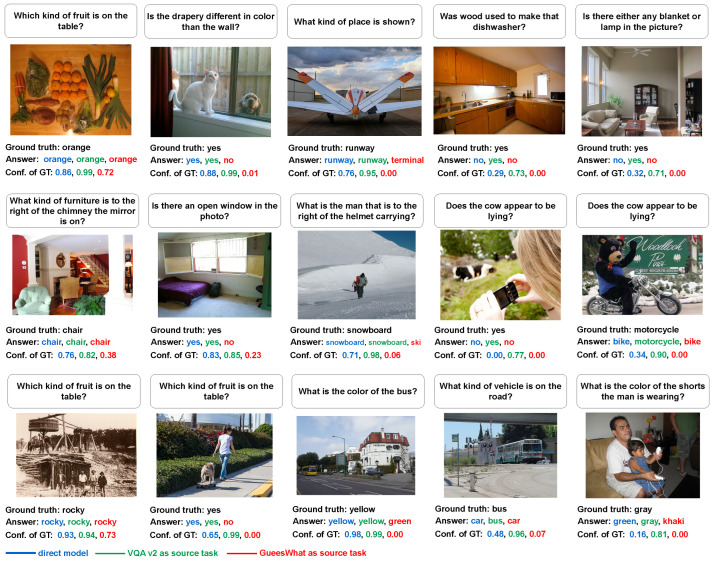
Example of the results on GQA. With the question on top of the image, different models predict the answer based on the image. The predictions from $m_{\mathrm{GQA}}$, $m_{\mathrm{VQA}\;v2\to \mathrm{GQA}}$, and $m_{\mathrm{GuessWhat}\to \mathrm{GQA}}$, as well as the confidence score of the ground truth class (Conf. of GT), are shown under the examples, respectively. It is easy to see that VQA v2 helps GQA to achieve a more accurate prediction, while GuessWhat misleads GQA to achieve a low confidence score in the ground truth class.

Figure 10 shows examples of the GQA validation set with the direct model $m_{GQA}$, one-hop model $m_{\mathrm{VQA}\;v2\to \mathrm{GQA}}$, and one-hop model $m_{\mathrm{GuessWhat}\to \mathrm{GQA}}$. We show the confidence of prediction for the ground truth class (Conf. of GT) under each example. VQA v2 gives the most positive effect to GQA, while GuessWhat gives the most negative effect. For example, in the second and third images from the left, GuessWhat induces wrong answers, whereas, in the last two images, VQA v2 helps to find the correct answers and improve the prediction with respect to the direct model.

## 6. Experiments on ViLT

In this section, we introduce our experiments on ViLT [75], which is also widely applied to multiple vision-and-language tasks (VQA v2 [10], COCO IR [9], Flickr30K IR [44], and NLVR2 [49]). To explore more tasks, we follow the instructions of ViLT and expand the model to support VG QA [43] and SNLI-VE [50]. Since ViLT directly uses the whole image (instead of the regions of the image) as the visual input, this model is not able to perform referring expression tasks. In general, we conduct a knowledge transferability exploration based on ViLT with six different vision-and-language tasks of three types:

- VQA: VQA v2 [10] and VG QA [43];
- IR: COCO IR [9] and Flickr30K IR [44];
- MV: NLVR2 [49] and SNLI-VE [50].

We follow the same experimental setting of ViLT (https://paperswithcode.com/method/vilt, accessed on 15 October 2024.) to fine-tune the model on each task and make the experiments with the same methodology in Section 4, i.e., train *direct* models $m_{t}$ and *one-hop* models $m_{s\to t}$, then compare their performance. We also use the same evaluation data and metrics as listed in Table 1.

### Main Observations

The experimental results are listed in Table 4. We have the following observations from these results:

**Observation** **1.** **Most of the tasks tend to obtain bad effects from other tasks.** Compared to the performance of the *direct* model $m_{t}$, the performance of *one-hop* models $m_{s\;\to \;t}$ in VQA v2, COCO IR, Flickr30K, and NLVR2 show significant decreases (more than 3%). These decreases may be related to catastrophic forgetting since ViLT is trained on more datasets than VilBERT. The result may indicate that catastrophic forgetting is one of the factors that affect knowledge transferability.
**Observation** **2.** **VG QA and SNLI-VE obtain help from some tasks to achieve better performance.** These results indicate that the knowledge from other vision-and-language tasks may still benefit models in solving some target tasks, even though catastrophic forgetting mitigates benefits.
**Observation** **3.** **SNLI-VE tends to obtain benefits from other tasks while having bad effects on them.** This observation is similar to VilBERT’s results in Observation 3 in Section 5.3, which indicates that the benefits of knowledge transferability are not reciprocal. The phenomenon may also indicate that the knowledge in SNLI-VE is different from other tasks, but other tasks may involve the knowledge for solving SNLI-VE.

## 7. Limitations and Future Work

*More complex transfer scenarios.* In this paper, we mainly focus on one-to-one transfer learning as it is the most widely used strategy in knowledge transferability. In our future work, we would like to make comprehensive explorations on other knowledge transfer strategies (e.g., more-to-more knowledge transfer scenario) in our future work.

*Optimal transfer point.* From our experiments, we observe that although GQA usually brings negative effects to other tasks, modifying the training setting of GQA during the pre-training step (e.g., decreasing the data scale of the GQA dataset or the training epochs of GQA) could make GQA a positive transfer to other tasks. These results may indicate that there is an optimal transfer point of knowledge transfer when the model learns just enough knowledge from the source task *s* but does not overfit. However, finding the optimal transfer point is a challenging task as the verification of the performance of model $m_{s\;\to \;t}$ needs another round of training and evaluation. We consider finding the optimal transfer point as one of our future work.

*Explorations on large-scale pre-training models.* Large-scale pre-training models, such as CLIP and BLIP-2, may contain rich and valuable knowledge that can bring more benefits to vision-and-language tasks. We are aware that it is important to involve the exploration based on these large-scale pre-training models. Actually, recent research has shown that studies [76,77] show that the knowledge from large-scale datasets is sensitive and easily suffers from catastrophic forgetting during the fine-tuning process. Since we focus more on the knowledge transferability between vision-and-language tasks, we would like to set the exploration of knowledge transferability from pre-text tasks or noisy but large-scale datasets as our future work.

## 8. Conclusions

We studied the knowledge transferability among 12 vision-and-language tasks. We confirmed that different tasks have different effects on each other, and the selection of tasks for knowledge transfer should be made carefully. Furthermore, we observed some interesting insights about knowledge transferability, e.g., the tasks in the image retrieval and referring expression groups tend to have a positive impact on other tasks, while the tasks in the visual question answering and multi-modal verification group give a negative contribution. The scale of datasets, training epochs, data domain similarity, and the difference in their goals may cause this divergence.

In general, this paper sheds light on the knowledge transferability of vision-and-language tasks, including those factors such as data scale and training epoch that may affect the transferability. We hope our work can bring inspiration to the fields of knowledge transferability in vision-and-language tasks, especially on the topic of seeking knowledge from similar tasks for positive transfer.

## Figures and Tables

**Table 1:** Dataset statistics for the 12 tasks used in our experiments. From the left, the first and second columns are the number of samples in the train and validation (Train +Val) set and test set, respectively. The third column is the metric to evaluate the corresponding task. The fourth column is the name of the test set. The fifth column is the number of images in the train and validation set. The last column is the source dataset from which the images of the corresponding task come from.

	Train + Val Samples	Test Samples	Evaluation Metric	Evaluation Set	Train + Val Image	Image Source
VQA v2 [10]	542,104	447,793	Accuracy	test-dev	98,861	MSCOCO [9]
VG QA [43]	1,294,255	5000	Accuracy	validation	92,147	MSCOCO [9] + YFCC100M [70]
GQA [18]	962,928	12,578	Accuracy	test-dev	69,868	Visual Genome [43]
COCO IR [9]	487,600	1000	Recall@5	test	99,435	MSCOCO [9]
Flickr30K IR [44]	140,485	1000	Recall@5	test	29,077	Flickr30K [51]
NLVR2 [49]	86,373	6967	Accuracy	test-P	29,808	NLVR2 [49]
SNLI-VE [50]	512,396	17,901	Accuracy	test	95,522	Flickr30K [51]
Visual7w [45]	93,813	57,265	Accuracy	test	16,415	MSCOCO [9]
GuessWhat [46]	100,398	23,785	Accuracy	test	51,291	MSCOCO [9]
refCOCO [47]	96,221	10,752	Accuracy	test	14,481	MSCOCO [9]
refCOCO+ [47]	95,852	10,615	Accuracy	test	14,479	MSCOCO [9]
refCOCOg [48]	65,514	9602	Accuracy	test	17,903	MSCOCO [9]

**Table 2:** Results of *direct* model $m_{t}$ in the 12 tasks.

10 Different Random Seeds		Avg ± Std	Max	Min
Task	VQA v2	70.3 ± 0.56	70.71	69.18
	VG QA (Val)	33.5 ± 0.48	34.17	32.86
	GQA	58.1 ± 0.53	58.65	57.10
	COCO IR	90.4 ± 0.77	91.02	89.12
	Flickr30K IR	86.5 ± 0.87	87.24	84.80
	NLVR2	73.4 ± 0.50	74.11	72.34
	SNLI-VE	75.3 ± 0.16	75.64	75.04
	Visual7w	80.4 ± 0.19	80.63	80.04
	GuessWhat	62.3 ± 0.17	62.68	62.14
	refCOCO	77.7 ± 0.30	78.15	77.13
	refCOCO+	69.1 ± 0.57	69.68	67.81
	refCOCOg	71.6 ± 0.63	72.50	70.50

**Table 3:** Knowledge transferability results per group. Results of $m_{row\;\to \;column}$ (rows 2–13, green or red color) are compared with the *direct* model $m_{column}$ (row 1, blue color) and assigned green (when the average score is higher than $m_{column}$, i.e., positive transfer) or red (when the average score is lower than $m_{column}$, i.e., negative transfer). Deep green/red shows the best/worst score in each column.

Avg ± Std		Target Task *t*											
		VQA v2	VG QA (Val)	GQA	COCO IR	Flickr30K IR	NLVR2	SNLI-VE	Visual7w	GuessWhat	refCOCO	refCOCO+	refCOCOg
*direct* model $m_{t}$		69.6 ± 0.71	33.7 ± 0.74	57.5 ± 0.59	89.2 ± 0.16	85.3 ± 0.48	72.8 ± 0.48	75.3 ± 0.11	80.1 ± 0.13	62.3 ± 0.08	77.3 ± 0.19	68.5 ± 0.72	70.8 ± 0.35
Source task *s*	VQA v2	-	33.5 ± 0.55	58.2 ± 0.21	89.0 ± 1.43	84.8 ± 1.04	73.7 ± 0.60	75.4 ± 0.23	79.5 ± 0.51	60.8 ± 0.62	76.8 ± 0.62	67.7 ± 1.12	70.5 ± 0.12
	VG QA	70.0 ± 0.33	-	57.5 ± 0.57	90.0 ± 0.11	84.3 ± 0.77	72.1 ± 0.69	75.7 ± 0.05	79.9 ± 0.91	60.9 ± 0.66	76.9 ± 0.57	68.0 ± 0.81	70.8 ± 0.33
	GQA	69.7 ± 0.16	33.0 ± 0.76	-	88.9 ± 1.17	82.9 ± 1.46	72.1 ± 0.76	75.3 ± 0.67	79.0 ± 0.12	60.7 ± 0.26	76.7 ± 0.32	67.2 ± 0.22	70.0 ± 0.42
	COCO IR	70.5 ± 0.56	33.6 ± 0.52	57.4 ± 0.49	-	86.8 ± 1.56	75.3 ± 0.24	76.1 ± 0.11	79.5 ± 0.34	62.0 ± 0.36	77.4 ± 0.59	69.4 ± 0.33	72.0 ± 0.25
	Flickr30K IR	70.3 ± 0.32	33.4 ± 0.69	57.6 ± 0.35	90.1 ± 1.30	-	74.0 ± 0.65	75.8 ± 0.22	79.8 ± 0.17	62.3 ± 0.16	77.3 ± 0.18	68.7 ± 0.22	71.3 ± 0.23
	NLVR2	69.9 ± 0.34	33.4 ± 0.35	57.5 ± 0.43	89.7 ± 1.16	84.8 ± 1.09	-	75.9 ± 0.06	79.4 ± 0.27	62.0 ± 0.17	77.1 ± 0.40	68.4 ± 0.40	70.9 ± 0.10
	SNLI-VE	69.9 ± 0.68	33.3 ± 0.51	57.3 ± 0.32	89.2 ± 0.51	85.5 ± 1.80	73.9 ± 0.24	-	79.2 ± 0.60	61.2 ± 0.40	76.8 ± 0.43	67.2 ± 0.41	70.4 ± 0.65
	Visual7w	70.2 ± 0.25	33.5 ± 0.94	57.7 ± 0.57	89.8 ± 0.45	85.6 ± 1.06	73.9 ± 0.71	76.1 ± 0.26	-	63.0 ± 0.36	78.1 ± 0.39	69.4 ± 0.06	72.8 ± 0.30
	GuessWhat	69.7 ± 0.61	33.5 ± 0.06	56.9 ± 0.33	89.5 ± 0.45	85.1 ± 2.09	73.2 ± 0.31	75.9 ± 0.34	80.8 ± 0.05	-	78.1 ± 0.13	69.1 ± 0.13	72.2 ± 0.06
	refCOCO	70.2 ± 0.22	33.7 ± 0.29	57.4 ± 0.21	90.1 ± 0.83	85.4 ± 1.33	73.7 ± 0.28	76.0 ± 0.33	80.3 ± 0.03	62.6 ± 0.29	-	69.5 ± 0.23	72.4 ± 0.29
	refCOCO+	70.1 ± 0.44	33.3 ± 0.05	57.2 ± 0.41	88.8 ± 1.93	84.9 ± 2.27	74.4 ± 0.22	76.1 ± 0.14	80.4 ± 0.17	62.5 ± 0.29	77.8 ± 0.35	-	73.0 ± 0.19
	refCOCOg	69.7 ± 0.30	34.0 ± 0.46	57.4 ± 1.07	89.1 ± 2.19	84.9 ± 1.24	74.1 ± 0.59	75.7 ± 0.20	80.5 ± 0.35	62.8 ± 0.15	78.3 ± 0.14	69.7 ± 0.35	-

**Table 4:** Knowledge transferability results in the ViLT model. Results of $m_{row\;\to \;column}$ (rows 2–7, green or red color) are compared with the *direct* model $m_{column}$ (row 1, blue color) and assigned green (when the average score is higher than $m_{column}$) or red (when the average score is lower than $m_{column}$). Deep green/red shows the best/worst score in each column.

ViLT		Target Task *t*					
		VQA v2	VG QA (Val)	COCO IR	Flickr30K IR	NLVR2	SNLI-VE
*direct* model $m_{t}$		71.32	35.10	93.10	88.80	76.55	72.58
Source task *s*	VQA v2	-	35.20	90.60	78.82	73.29	73.06
	VG QA	69.48	-	89.24	76.26	70.34	72.29
	COCO IR	71.10	34.99	-	79.74	72.31	72.94
	Flickr30K IR	69.93	34.79	89.14	-	73.59	73.28
	NLVR2	70.87	34.66	89.46	78.20	-	72.67
	SNLI-VE	65.97	33.25	85.46	74.60	63.46	-

## Data Availability

No new data were created in this paper.
